# The outcome of hydrodilation in frozen shoulder patients and the relationship with kinesiophobia, depression, and anxiety

**DOI:** 10.1186/s40634-021-00394-3

**Published:** 2021-09-30

**Authors:** Philippe Debeer, Olivia Commeyne, Ianthe De Cupere, Dorien Tijskens, Filip Verhaegen, Wim Dankaerts, Laurence Claes, Glenn Kiekens

**Affiliations:** 1 Orthopedics, University Hospitals Leuven, Herestraat 49, B-3000 Leuven, Belgium; 2 Department of Development and Regeneration, KU Leuven, Leuven, Belgium; 3 Institute for Orthopaedic Research and Training, Leuven, Belgium; 4 Musculoskeletal Research Unit, Department of Rehabilitation Sciences, Faculty of Kinesiology and Rehabilitation Sciences, KU Leuven, Leuven, Belgium; 5 Faculty of Psychology and Educational Sciences, KU Leuven, Leuven, Belgium; 6 Faculty of Medicine and Health Sciences, University Antwerp, Antwerp, Belgium; 7 Department of Neurosciences, Center for Contextual Psychiatry, KU Leuven, Leuven, Belgium

**Keywords:** Frozen shoulder, Depression, Anxiety

## Abstract

**Purpose:**

The aims of this study were to (1) investigate the effect of hydrodilatation in frozen shoulder patients on objective indices of shoulder functionality and subjective outcomes of pain, mobility, kinesiophobia, depression, and anxiety, and (2) progress knowledge about the reciprocal temporal relationship between psychological parameters at baseline and objective and subjective outcomes at 3-month follow-up.

**Methods:**

We evaluated the clinical and psychological status of 72 patients with a frozen shoulder before and after hydrodilatation, using the Constant Murley score, the Visual Analogue score, the Tampa Scale for Kinesiophobia, the Hospital Anxiety and Depression Scale, and the Shoulder Pain And Disability Index.

**Results:**

We noted a significant improvement in functionality, pain and disability (*p* < .001). Depression and anxiety improved significantly (*p* < .001) between baseline and 3-month follow-up. Prospective analyses demonstrated that psychological factors are more likely to predict outcomes of hydrodilatation than vice versa.

**Conclusion:**

Hydrodilatation followed by physiotherapy is an excellent way to treat patients with recalcitrant frozen shoulder, resulting in a continuous improvement of ROM and pain. Physiotherapists and physicians should be aware that psychological factors might have an impact on the treatment outcome.

## Background

Psychosocial factors are frequently present in patients with shoulder complaints and might play a role in the disease, the perception of pain and disability, and the functional outcome after surgery [16]. The chronicity of the complaints often negatively impacts the quality of life and physical and mental well-being. Kinesiophobia, negative pain belief, low pain efficacy, and catastrophizing can be associated with higher levels of pain and disability in the upper extremity problems [9, 25]. Similarly, depression has been associated with worse surgical outcomes in several musculoskeletal conditions and seems to be more prevalent in orthopaedic patients than in the general population [8]. More specifically, in patients with shoulder problems, depression and anxiety can negatively influence health-related quality of life and are associated with higher levels of functional disability and a longer duration of symptoms [5, 22]. Some studies demonstrate worse postoperative outcomes in patients with anxiety and depression, whereas others found no influence of these psychological parameters after shoulder surgery [6, 11, 17, 19].

This study focuses on patients with frozen shoulder, a disabling condition of the shoulder characterized by often severe pain and functional restriction of both active and passive shoulder motion for which radiographs of the glenohumeral joint are essentially unremarkable. Although some patients with a frozen shoulder improve without any intervention, the majority of patients continue to have pain and/or restriction of mobility [26]. Similar observations were made by Abrassart et al. [1]. These authors concluded that the natural history of frozen shoulder often sees short-term improvement but bears a high chance of ongoing low-level restriction and pain. The condition can be primary or idiopathic, or secondary, indicating a specific cause [28]. Treatment options for frozen shoulder include physiotherapy, injections, hydrodilatation, manipulation under anesthesia, and capsular release. Hydrodilatation consists of injecting fluid in the glenohumeral joint under fluoroscopic control to rupture the capsule to increase the shoulder’s mobility [2]. It is an easy, safe, and cost-effective method to treat frozen shoulder in terms of functionality and pain relief [4, 14, 15, 20, 21, 27]. Moreover, when a first hydrodilatation does not result in adequate pain relief and/or restoration of mobilty, the procedure can easily be repeated before proceeding to a surgical procedure.

Clinicians often have the impression that patients with a frozen shoulder have a specific personality and tend to be neurotic, tense and have a low pain threshold. In a previous study, we could not confirm the assertion that such a ‘frozen shoulder personality’ exists [10]. Several studies found that psychosocial factors like depression, anxiety, and kinesiophobia are frequently associated with frozen shoulder [12, 13]. While these studies provide meaningful information about the mental health comorbidity of patients with a frozen shoulder, they are all cross-sectional, which limits the conclusions we can draw about the directionality of effects (i.e., whether depression and anxiety predict objective and subjective frozen shoulder outcomes, or whether these outcomes increase the risk of worse mental health problems, or both).

The present study has two primary aims. First, we wanted to confirm the beneficial effect of hydrodilatation in frozen shoulder patients on objective indices of shoulder functionality and subjective outcomes of pain, mobility, kinesiophobia, depression, and anxiety. We hypothesized that a hydrodilatation would increase the shoulder’s functionality, decrease the pain and that the psychological parameters would negatively influence the outcome. Second, we investigated whether anxiety, depression, and kinesiophobia at baseline negatively predict the objective and subjective outcomes of hydrodilatation at 3-month follow-up, and that worse objective and subjective outcomes of hydrodilation positively predict worse mental health complaints at 3-month follow-up. Specifically, we hypothesized that higher levels of kinesiophobia, depression, and anxiety would attenuate the objective and subjective outcomes of hydrodilation, and vice versa, that improvement in objective and subjective clinical parameters would lead to less kinesiophobia, depression, and anxiety at three-month follow-up.

## Methods

### Participants

We prospectively enrolled patients who presented to our Orthopaedic Upper Limb Clinic with a frozen shoulder between September 2014 and January 2018. The diagnosis of frozen shoulder was made on clinical grounds. Based on the criteria of Zuckerman and Rokito**,** patients were further subdivided into a primary and a secondary frozen shoulder group [28]. All participants provided written informed consent before inclusion in the study. This study was approved by the Full Local Research and Ethical Committee of the University Hospital of Leuven (B322201421602; S56800).

### Assessments and instruments

The active and passive mobility of both the affected and unaffected shoulder was recorded using a goniometer with the patient standing. Active internal rotation at the back was measured by recording the vertebral level reached with the thumb’s tip. This level was serially numbered 1 to 12 for T1 to T12; 13 to 17 for L1 to L5, and 18 for any level below the sacral region.

All patients had radiographs of the shoulder to detect any bony abnormalities or rotator cuff calcifications. We used ultrasound and magnetic resonance to evaluate any other underlying pathology. Exclusion criteria were stiffness caused by glenohumeral osteoarthritis, reflex sympathetic dystrophy of the ipsilateral hand, stiffness after shoulder arthroplasty, malignant neoplasms of the shoulder girdle, and mental incapacity to fill in the questionnaires.

### Clinical assessment

Functionality of the shoulder was assessed using the *Constant-Murley Score* (CMS) [7]. The *Shoulder Pain And Disability Index (SPADI)* was used to measure pain and disability [23]**.** The SPADI is a self-report questionnaire and contains two domains: pain (five items) and disability (eight items). All patients completed the *Hospital Anxiety and Depression Scale (HADS)* to determine anxious and depressive symptomatology. The HADS assesses anxiety and depression in non-psychiatric patients, with higher scores indicating higher levels of anxiety and depression symptoms [3]. We used the short version of the *Tampa Scale for Kinesiophobia (TSK-11)* to evaluate the degree of kinesiophobia. Higher scores indicate higher levels of kinesophobia [24]. The pain level was assessed with the *Visual analogue Score (VAS).*

All patients were examined clinically before the hydrodilatation (time-point T1), immediately after the hydrodilatation (time-point T2), and three months after hydrodilatation (time-point T3). The VAS, HADS, SPADI and TSK-11 were completed at T1 and T3.

### Hydrodilatation

At our institution, hydrodilatation is prescribed by the orthopaedic surgeon (PD, FV) for patients with adhesive capsulitis with persistent pain and stiffness. A musculoskeletal radiologist performed hydrodilatation under fluoroscopic guidance with the patient supine on the table. A 21-gauge needle was inserted in the glenohumeral joint, the intra-articular position was confirmed with 5 mL contrast medium (Ultravist) and a mixture of 40 mg methylprednisolonacetate, 15 mL of local anesthetic (marcaine 0.5%), and 20 mL of normal saline was slowly injected until the capsule was ruptured, as demonstrated on the fluoroscopy monitor by extracapsular leakage of the contrast medium. A physiotherapist recorded the active and passive mobility of the shoulder at T1 and T2. All patients received a standardized physiotherapy protocol to perform under the supervision of their local physiotherapist. The protocol typically consists of gentle active and passive shoulder mobilization exercises within the tolerated range together with stretching exercises for the chest muscles and muscles at the back of the shoulder. Aggressive exercises should be avoided, because they can worsen the capsular synovitis and subsequently cause pain. Strengthening exercises for the rotator cuff, the deltoid, the chest muscles and the scapulothoracic musculature can be started when the range of motion is gradually improving.

### Statistical analysis

We used the expectation–maximization method to account for missing data (max 5 cases/variable). To evaluate the potential beneficial effect of hydrodilatation on objective and subjective outcomes (objective one), we calculated change scores of active and passive ROM, perceived pain, disability associated with shoulder pathology, depression, anxiety, and kinesiophobia. Significant changes over time for each of these variables were assessed using repeated measure ANOVAs with time as a within-subject variable. In a second step, we evaluated the moderating role of age, sex, and type of frozen shoulder in a multivariate model to clarify the generality of the effects of hydrodilatation. These effects were considered significant at α = 0.05.

We performed multivariate linear regressions to investigate the reciprocal temporal effects between objective and subjective outcomes of hydrodilatation on the one hand, and depression, anxiety, and kinesiophobia on the other hand (objective two). These analyses predicted clinical outcomes at T3 and included each time the dependent variable's score at pre-assessment, age, sex, and frozen shoulder type. This method allowed us to investigate each independent variable's incremental predictive effect on each outcome of interest. We used 95% bias-corrected and accelerated bootstrap confidence intervals to identify significant predictors. All analyses were performed using SPSS version 26.

## Results

A total of 72 patients, 44 women and 28 men with a mean age of 53 years (*SD* = 7, range, 38–70 years) were enrolled in this study. The median time that patients had complaints was eight months (Interquartile range = 5.3–12.0). Twenty-five patients had an idiopathic frozen shoulder. The group of secondary frozen shoulders (*n* = 47) was very heterogeneous. Nine patients had diabetes mellitus, 13 had thyroid problems, 20 had hypercholesterolemia or a combination. A postoperative frozen shoulder was noted after arthroscopic stabilization (*n* = 1), arthroscopic release of frozen shoulder (*n* = 2), arthroscopic rotator cuff repair (*n* = 2), arthroscopic needling of calcifications (*n* = 3), and arthroscopic decompression (*n* = 2). Eleven patients had a frozen shoulder associated with calcific tendinitis; 4 had a partial rotator cuff tear, one had pulmonary disease, and two had cardiac disease. One patient had stiffness after a shoulder dislocation, and 13 patients developed stiffness after minor trauma (contusion, fall on the shoulder without fracture). Twenty-six patients were not previously treated for their frozen shoulder. Forty-four patients had undergone previous non-operative treatment consisting of infiltrations (*n* = 28), physiotherapy (*n* = 23), failed hydrodilatation (*n* = 1). Two patients presented with a persistent frozen shoulder after a failed arthroscopic release.

### Objective One: Temporal effects between hydrodilatation and objective indices of shoulder functionality and subjective outcomes of pain, mobility, kinesiophobia, depression, and anxiety

The CMS shoulder increased significantly (*p* < 0.001) from 46.2 (SD = 13.0) at T1 to 72.0 (SD = 16.9) at T3, indicating an average improvement of 25.8 points (95%CI = 21.8–29.8). Table 1 displays the changes in shoulder functionality indices at different time points. All mobility parameters show a significant (*p* < 0.001) improvement from T1 to T2 and T3. Pain and disability, as indicated by the SPADI, decreased significantly (*p* < 0.001) from 33.1 (SD = 9.6) and 48.6 (SD = 17.7) to 18.0 (SD = 13.0) and 23.0 (SD = 21.7), respectively (mean reduction pain = 15.9, 95%CI = 12.4–17.8, mean reduction disability = 25.6, 95%CI = 21.4–29.9). Females reported a stronger decrease in pain (17.5 versus 10.8, *p* = 0.016) and mobility scores (29.1 versus 19.0, *p* = 0.023) on the SPADI than males. The VAS pain score decreased significantly (*p* < 0.001) from 5.3 (SD 2.4) at T1 to 2.4 (SD = 2.8) at T3 (mean reduction 2.9, 95%CI = 2.3–3.5). The type of frozen shoulder did not influence these effects.

**Table 1:** Univariate changes in indices of shoulder functionality parameters at different time-points

Functional parameter	T_1 (pre)_		T_2 (post)_		T_3 (3 months post)_		F^a^	Significant pairwise comparisons
	*M*	*SD*	*M*	*SD*	*M*	*SD*		
Active abduction	94.8	26.8	114.2	35.2	150.1	35.7	61.6***	T_1_ < T_2_,T_1_ < T_3_,T_2_ < T_3_
Active anteflexion	112.6	19.8	128.4	27.6	158.0	23.8	83.2***	T_1_ < T_2_,T_1_ < T_3_,T_2_ < T_3_
Active external rotation	30.5	14.0	47.2	13.7	51.9	17.4	60.1***	T_1_ < T_2_,T_1_ < T_3_,T_2_ < T_3_
Active internal rotation	16.0	3.0	14.1	4.7	9.3	6.0	47.7 ***	T_1_ > T_2_,T_1_ > T_3_,T_2_ > T_3_
Passive abduction	81.3	25.3	96.7	33.1	99.1	23.0	18.7***	T_1_ < T_2_,T_1_ < T_3_
Passive anteflexion	103.3	22.2	118.0	27.0	121.8	26.1	20.2***	T_1_ < T_2_,T_1_ < T_3_
Passive external rotation	26.7	13.6	41.0	15.1	38.1	16.2	37.9***	T_1_ < T_2_,T_1_ < T_3_
Passive internal rotation	32.8	23.2	52.6	17.6	53.1	24.4	25.6***	T_1_ < T_2_,T_1_ < T_3_

Note: *T1* before HD, *T2* immediately after HD, *T3* 3 months after HD, *M* Mean, *SD* Standard Deviation

^a^ F-test based on the independent pairwise comparisons among the estimated marginal means

^***^p < 0.001

Patients with a longer duration of symptoms reported higher levels of pain and disability on the SPADI (*r* = 0.29-0.30 range) and VAS (*r* = 0.26) at T1 and less favorable change at T3 on the CMS and VAS. Depression (mean reduction 1.5, 95%CI = 0.9–2.1), and anxiety (mean reduction 1.1, 95%CI = 0.5–1.7) improved significantly (*p* < 0.001) between T1 and T3. Kinesiophobia improved significantly for females (mean reduction 3.0, 95%CI = 2.0–4.1, *p* < 0.001), but not for males (95%CI = -1.7–3.1, *p* = 0.568).

### Objective Two: To investigate the reciprocal temporal effects between objective and subjective outcomes of hydrodilatation and kinesiophobia, depression, and anxiety

When considering reciprocal effects between the examined outcomes of hydrodilatation and respectively, kinesiophobia (Table 2), depression (Table 3), and anxiety (Table 4), psychological factors were more likely to predict outcomes of hydrodilatation than vice versa. Specifically, kinesiophobia, depression, and anxiety at T1 significantly predicted worse outcomes at T3 on the VAS and active abduction. Kinesiophobia and depression also predicted a lower CMS and active anteflexion score, whereas depression and anxiety were predictive of a higher residual disability index at T3.

**Table 2:** The reciprocal effects between objective and subjective outcomes of hydrodilatation and kinesiophobia

Predictor at t1	*β*	95% BCI	Outcome at t3
Objective and subjective outcomes at 3-month follow-up			
CMS total	0.37*	.08; 0.70	CMS Total
Kinesiophobia	-0.89*	-1.44;-0.34	
SPADI_Pain	0.62*	0.33; 0.91	SPADI_Pain
Kinesiophobia	0.58*	0.13; 1.01	
SPADI_Disability	0.66*	0.43; 0.90	SPADI_Disability
Kinesiophobia	0.59	-0.21;1.33	
VAS_Pain	0.48*	0.25; 0.72	VAS_Pain
Kinesiophobia	0.10*	0.02; 0.17	
Active abduction	0.03	-0.21; 0.27	Active Abduction
Kinesiophobia	-2.30*	-3.52; -1.33	
Active Anteflexion	0.07	-0.21; 0.37	Active Anteflexion
Kinesiophobia	-1.28*	-2.04; -0.61	
Active external rotation	0.31*	0.01; 0.64	Active External Rotation
Kinesiophobia	-0.74*	-1.29; -0.27	
Active internal rotation	0.38	-0.02; 1.00	Active Internal Rotation
Kinesiophobia	0.39*	0.18; .0.58	
Passive abduction	-0.08	-0.31; -0.22	Passive Abduction
Kinesiophobia	-1.00*	-1.85; -0.09	
Passive Anteflexion	0.23	-0.15; 0.63	Passive Anteflexion
Kinesiophobia	0.3	-0.89; 1.61	
Passive external rotation	0.35	-0.02; 0.66	Passive External Rotation
Kinesiophobia	-0.43	-0.92; 0.04	
Passive internal rotation	0.31*	0.01; 0.64	Passive Internal Rotation
Kinesiophobia	-0.42	-1.04; 0.22	
Psychological outcomes at 3-month follow-up			
CMS Total	-0.01	-.11; .09	Kinesiophobia
Kinesiophobia	0.92*	0.73; 1.07	
SPADI_Pain	0.09	-0.05; 0.24	Kinesiophobia
Kinesiophobia	0.89*	0.67; 1.06	
SPADI_Disability	0.02	-0.05; 0.09	Kinesiophobia
Kinesiophobia	0.90*	0.70; 1.08	
VAS_Pain	0.20	-0.27; 0.66	Kinesiophobia
Kinesiophobia	0.90*	0.71; 1.07	
Active Abduction	0.06*	0.02; 0.10	Kinesiophobia
Kinesiophobia	0.98*	0.80; 1.15	
Active Anteflexion	0.03	-0.05; 0.10	Kinesiophobia
Kinesiophobia	0.95*	0.79; 1.10	
Active External Rotation	0.03	-0.04; 0.11	Kinesiophobia
Kinesiophobia	0.93*	0.75; 1.08	
Active Internal Rotation	-0.29*	-0.54; -0.04	Kinesiophobia
Kinesiophobia	0.95*	0.77;1.12	
Passive Abduction	0.02	-0.04; 0.07	Kinesiophobia
Kinesiophobia	0.94*	0.76; 1.09	
Passive Anteflexion	0.02	-0.03; 0.07	Kinesiophobia
Kinesiophobia	0.93*	0.75; 1.09	
Passive External Rotation	0.03	-0.05; 0.12	Kinesiophobia
Kinesiophobia	0.92*	0.74; 1.09	
Passive Internal Rotation	0.01	-0.04; 0.08	Kinesiophobia
Kinesiophobia	0.93*	0.75; 1.07	

*Note*: Significance is based on 95% bias-corrected and accelerated bootstrap confidence intervals (BCI). All regressions controlled for age, sex, and type of frozen shoulder (not shown in this table for ease of interpretation)

**Table 3:** The reciprocal effects between objective and subjective outcomes of hydrodilatation and depression

Predictor at t1	*β*	95% BCI	Outcome at t3
Objective and subjective outcomes at 3-month follow-up			
CMS total	0.30*	0.01; 0.61	CMS Total
Depression	-1.74*	-3.16; -0.20	
SPADI_Pain	0.65*	0.41; 0.91	SPADI_Pain
Depression	0.67	-0.10; 1.51	
SPADI_Disability	0.63*	0.43; 0.84	SPADI_Disability
Depression	1.29*	0.09; 2.48	
VAS_Pain	0.48*	0.28. 0.69	VAS_Pain
Depression	0.18*	0.04. 0.32	
Active abduction	0.03	-0.22. 0.28	Active Abduction
Depression	-2.95*	-5.26. -0.81	
Active Anteflexion	0.09	-0.19; 0.40	Active Anteflexion
Depression	-1.55*	-3.37; -0.04	
Active external rotation	0.29	-0.02; 0.59	Active External Rotation
Depression	-0.65	-1.78; 0.45	
Active internal rotation	0.49*	0.01; 1.08	Active Internal Rotation
Depression	0.37	-0.04; 0.72	
Passive abduction	-0.13	-0.34; 0.16	Passive Abduction
Depression	-2.05	-3.60; 0.14	
Passive anteflexion	0.10	-0.30; 0.49	Passive Anteflexion
Depression	-2.31*	-4.02; -0.63	
Passive external rotation	0.31	-0.05; 0.63	Passive External Rotation
Depression	-0.51	-1.37; 0.55	
Passive internal rotation	0.30	-0.02; 0.640	Passive Internal Rotation
Depression	-1.35*	-2.44; -0.04	
Psychological outcomes at 3-month follow-up			
CMS Total	0.00	-0.06; 0.05	Depression
Depression	0.72*	0.51; 0.88	
SPADI_Pain	0.03	-0.04; 0.10	Depression
Depression	0.70*	0.49; 0.84	
SPADI_Disability	0.01	-0.03. 0.05	Depression
Depression	0.71*	0.49. 0.86	
VAS_Pain	-0.02	-0.25. 0.23	Depression
Depression	0.73*	0.52. 0.86	
Active abduction	0.00	-0.02; 0.03	Depression
Depression	0.73*	0.52; 0.86	
Active anteflexion	0.02	-0.01; 0.05	Depression
Depression	0.75*	0.53; 0.88	
Active external rotation	-0.01	-0.05; 0.04	Depression
Depression	0.72*	0.52; 0.86	
Active internal rotation	-0.20	-0.42; 0.01	Depression
Depression	0.74*	0.54; 0.87	
Passive abduction	-0.01	-0.03; 0.00	Depression
Depression	0.70*	0.48; 0.84	
Passive anteflexion	0.00	-0.02; 0.02	Depression
Depression	0.73*	0.50; 0.86	
Passive external rotation	0.03	-0.02; 0.09	Depression
Depression	0.76*	0.55; 0.09	
Passive internal rotation	-0.01	-0.03; 0.02	Depression
Depression	0.72*	0.52; 0.86	

*Note*: Significance is based on 95% bias-corrected and accelerated bootstrap confidence intervals (BCI). All regressions controlled for age, sex, and type of frozen shoulder (not shown in this table for ease of interpretation)

**Table 4:** The reciprocal effects between objective and subjective outcomes of hydrodilatation and anxiety

Predictor at t1	*β*	95% BCI	Outcome at t3
Objective and subjective outcomes at 3-month follow-up			
CMS total	0.44*	0.12; 0.77	CMS Total
Anxiety	-1.11	-2.35; 0.23	
SPADI_Pain	0.72*	0.50; 0.98	SPADI_Pain
Anxiety	0.57	-0.34; 1.40	
SPADI_Disability	0.72*	0.53; 0.90	SPADI_Disability
Anxiety	1.27*	0.03; 2.31	
VAS_Pain	0.54*	0.35; 0.75	VAS_Pain
Anxiety	0.14*	0.01; 0.28	
Active abduction	0.07	-0.19; 0.33	Active Abduction
Anxiety	-3.41*	-5.58; -1.43	
Active anteflexion	0.14	-0.15; 0.45	Active Anteflexion
Anxiety	-1.29	-2.77; 0.06	
Active external rotation	0.31	-0.00; 0.62	Active External Rotation
Anxiety	-0.35	-1.66; 1.11	
Active internal rotation	0.51*	0.04; 1.14	Active Internal Rotation
Anxiety	0.24	-0.18; 0.60	
Passive abduction	-0.13	-0.36; 0.18	Passive Abduction
Anxiety	-2.22	-4.15; 0.39	
Passive anteflexion	0.16	-0.23; 0.56	Passive Anteflexion
Anxiety	-1.77	3.57; 0.14	
Passive external rotation	0.34	-0.04; 0.67	Passive External Rotation
Anxiety	-0.46	-1.52; 0.73	
Passive internal rotation	0.30	-0.02. 0.63	Passive Internal Rotation
Anxiety	-0.63	-1.94. 0.76	
Psychological outcomes at 3-month follow-up			
CMS total	-0.02	-0.07; 0.04	Anxiety
Anxiety	0.77*	0.59; 0.93	
SPADI_Pain	0.03	-0.03; 0.10	Anxiety
Anxiety	0.77*	0.60; 0.93	
SPADI_Disability	0.02	-0.01; 0.05	Anxiety
Anxiety	0.77*	0.59; 0.93	
VAS_Pain	0.09	-0.16; 0.036	Anxiety
Anxiety	0.78*	0.60; 0.94	
Active abduction	0.00	-0.03; 0.02	Anxiety
Anxiety	0.78*	0.60; 0.93	
Active anteflexion	-0.01	-0.04; 0.02	Anxiety
Anxiety	0.77*	0.59; 0.94	
Active external rotation	0.02	-0.02; 0.07	Anxiety
Anxiety	0.79*	0.60*0.96	
Active internal rotation	-0.13	-0.34; 0.07	Anxiety
Anxiety	0.79*	0.61; 0.96	
Passive abduction	-0.01	-0.03; 0.01	Anxiety
Anxiety	0.76*	0.56; 0.94	
Passive anteflexion	-0.02	-0.04; 0.01	Anxiety
Anxiety	0.76*	0.56; 0.93	
Passive external rotation	0.03	-0.02; 0.09	Anxiety
Anxiety	0.79*	0.61; 0.96	
Passive internal rotation	0.04*	0.01. 0.07	Anxiety
Anxiety	0.82*	0.64. 0.99	

*Note*: Significance is based on 95% bias-corrected and accelerated bootstrap confidence intervals (BCI). All regressions controlled for age, sex, and type of frozen shoulder (not shown in this table for ease of interpretation

Several psychological factors were also uniquely related to the outcomes of hydrodilatation. Kinesiophobia at T1 increased the probability of a less positive effect of hydrodilatation on subjective pain, assessed with the SPADI, and the shoulder functionality indices active external rotation, active internal rotation, and passive abduction. Depression also predicted worse passive anteflexion and internal rotation scores at T3. Conversely, none of the objective and subjective measures at T1 significantly predicted depression at T3. Furthermore, of all the assessed prospective associations, only active abduction and active and passive internal rotation were significantly predictive of kinesiophobia and/or anxiety.

## Discussion

This is the first prospective study to investigate the associations between kinesiophobia, depression, and anxiety and clinical outcomes of a validated treatment option in frozen shoulder patients. Our study seems to confirm the good short-term functional scores after hydrodilatation in patients with frozen shoulder. A fast improvement of the ROM is seen together with a decrease of pain levels. This is in concordance with a study by Yoon et al. [27]. In their series, hydrodilatation also yielded a rapid improvement of mobility and decreased pain after three months. However, after six months, no significant differences were noted between hydrodilatation and subacromial or intra-articular corticosteroid injection. The rapid treatment effect is the main reason why hydrodilatation is our preferred non-operative treatment option for frozen shoulder. Most frozen shoulder patients already suffer for a long time, and a direct improvement in pain and mobility is often a stimulus to continue physiotherapy to regain a normal ROM. Here, a longer duration of symptoms was associated with a lower functional result. This can be explained by the fact that patients with long-standing mobility impairment probably have a very thickened glenohumeral capsule that withstands hydrodilatation. In general, the good functional result after hydrodilatation also resulted in the better psychological well-being of frozen shoulder patients, with significantly lower levels of pain, anxiety, and depression. However, a longer duration of symptoms negatively influenced the psychological outcome with higher levels of kinesiophobia, more anxiety/depression, and more pain/disability three months after the hydrodilatation.

The most important aim of this study was to investigate the reciprocal temporal effects between objective and subjective outcomes of hydrodilatation and kinesiophobia, depression, and anxiety. We hypothesized that hydrodilatation would lead to a decrease in subjective outcomes of pain and an increase in mobility, but such that higher levels of depression and/or anxiety would attenuate this improvement. This was especially true for kinesiophobia and depression. In addition, we anticipated that an improvement in objective and subjective clinical parameters would lead to less kinesiophobia, depression, and anxiety at a three-month follow-up assessment, but this was generally not confirmed. Koorevaar et al. also noted that psychological symptoms persisted in 56% of patients after surgical treatment [17]. Similarly, they found that psychological disorders one year postoperatively were associated with a worse clinical outcome. Our results suggest that improvement in psychological factors may improve the outcomes of hydrodilation but did not found evidence for the reverse direction. The latter suggests that mental health improvement is likely driven by more factors than those measured in this study.

### Study limitations

The current findings should be interpreted in the context of several limitations. First, we only evaluated the possible effect of psychological factors on the outcome of one type of treatment for frozen shoulder and did not consider prior treatments. Since this is a referral hospital, most frozen shoulder patients have already run through a physiotherapy and/or injections program, so the beneficial effect of the hydrodilatation might also be partially attributed to the previous therapy. However, this effect is probably minimal, as patients would otherwise not have sought further treatment. Further, the fact that the patients did not have the same physiotherapist to perform the prescribed rehabilitation at home might have created a bias. However, it was not practically possible to provide supervised physiotherapy with the same physiotherapist in one center in our clinical setting. Second, as we did not have a control group, findings regarding treatment outcomes of hydrodilatation should be interpreted tentatively. Relatedly, we used convenience sampling of patients who presented to our Orthopaedic Upper Limb Clinic with a frozen shoulder between September 2014 and January 2018 while the sample size was not predetermined based on apriori power analyses. Hence, future research is needed to evaluate the generalizability and replicability of the present findings. Third, although it is a clinical reality that the group frozen shoulders are heterogeneous, this might be a confounding factor regarding in our analyses of treatment outcomes and its relationship with psychological parameters. Furthermore, it should be acknowledged that the sample size in the present study was too low to conduct any meaningful subgroup analyses of lagged effects between patients with primary and secondary frozen shoulder and those with and without a prior treatment history. This represents an important direction for future research. Fourth, the follow-up time after hydrodilatation was only three months. Consequently, we cannot comment on the long-term effects of this treatment. However, Fareed et al. reported sustainable effects up to ten years after hydrodilatation [14]. Similarly, Nicholson et al. observed low repeat intervention rates after hydrodilatation at long-term follow-up [18]. Fifth, some patients had a duration of symptoms of less than six months. These would be classified in the frozen shoulder’s inflammatory stage, whereas those with a longer duration of symptoms would be in the freezing or frozen phase. One could argue that a hydrodilatation in the early inflammatory phase would be too painful for the patients. However, in our experience, the presence of a local anesthetic and corticosteroids in the hydrodilatation cocktail provides enough pain relief in these patients. Finally, we used self-reported scores to evaluate the psychological well-being of our patients. Social desirability bias can be an issue when patients want to picture themselves in a socially more acceptable manner.

## Conclusion

We observed a significant improvement in functionality, pain, and disability following hydrodilatation and found that kinesiophobia, depression, and anxiety predict the treatment outcome of hydrodilatation on frozen shoulder symptomatology. Awaiting future research on this timely topic, the present finding suggests that knowledge of frozen shoulder patients’ psychological well-being might help guide the most suitable treatment strategy.

## Data Availability

The datasets generated and analyzed during the current study are available from the authors on reasonable request.
